# Clinical Screening Tools to Diagnose Group A Streptococcal Pharyngotonsillitis in Primary Care Clinics to Improve Prescribing Habits

**DOI:** 10.21315/mjms2018.25.6.2

**Published:** 2018-12-28

**Authors:** Abdulrahman Muthanna, Hani Syahida Salim, Rukman Awang Hamat, Nurainul Hana Shamsuddin, Siti Zulaikha Zakariah

**Affiliations:** 1 Department of Medical Microbiology and Parasitology, Faculty of Medicine and Health Sciences, Universiti Putra Malaysia, 43400 UPM Serdang, Selangor, Malaysia; 2 Department of Family Medicine, Faculty of Medicine and Health Sciences, Universiti Putra Malaysia, 43400 UPM Serdang, Selangor, Malaysia

**Keywords:** pharyngitis, clinical score, Centor score, Streptococcus pyogenes, antibiotic prescription

## Abstract

This review highlights the clinical scoring tools used for the management of acute pharyngotonsillitis in primary care clinics. It will include the prevalence of group A pharyngotonsillitis among children and adults worldwide and the selective tests employed for diagnosing group A streptococcal pharyngotonsillitis. Pharyngotonsillitis is one of the common reasons for visits to primary care clinics worldwide, and physicians tend to prescribe antibiotics according to the clinical symptoms, which leads to overprescribing antibiotics. This in turn may lead to serious health impacts and severe reactions and may promote antibiotic resistance. These significantly add on to the health care costs. The available information from health organisations and previous studies has indicated the need to manage the diagnosis of pharyngotonsillitis to improve prescribing habits in primary care clinics.

## Introduction

Acute pharyngotonsillitis is the second most commonly diagnosed pediatric illness, with sporadic cases among adults (1). Group A streptococcal (GAS) infection frequently causes significant morbidity and is associated with significant mortality rates worldwide. Around the world, more than 600 million cases annually have been diagnosed as pharyngotonsillitis (1). Serious complications of pharyngotonsillitis caused by *Streptococcus pyogenes* (also known as group A streptococcus) are rheumatic fever, scarlet fever, toxic shock syndrome, and acute glomerulonephritis (2). *Streptococcus pyogenes* is responsible for about 15%–30% and about 5%–10% of acute pharyngotonsillitis cases in children and adults, respectively (1). Clinical signs and symptoms alone cannot be used to rule out or diagnose pharyngotonsillitis, as it can mimic other types of diagnosed infections (3). Thus, diagnosis of pharyngotonsillitis has always been delayed. Ideally, the diagnosis of GAS pharyngotonsillitis should be confirmed by throat swab culture, which usually takes two to three days for the bacterial growth to be identified. During this period, the illness might be resolved, or patients might experience several complications (1). Viral pharyngotonsillitis is treated by a symptomatic relief, whereas GAS pharyngotonsillitis may require the prescription of antibiotics such as penicillin, clindamycin, or erythromycin. A specific treatment might be required if complications occur (4, 5). In general, antibiotics are safe but should be prescribed by a physician after a careful clinical assessment. However, antibiotics that are taken unnecessarily may contribute to the development of antibiotic resistance (6). The excessive use of antibiotics and an unnecessary antibiotic prescription add an economic burden to the health care system worldwide, as well as to patients and their families (6). Studies have shown trends of inappropriate prescribing of antibiotics for upper respiratory tract infection (URTI) in Malaysian primary care, where antibiotic prescribing rates for URTI were 46.7% in Malaysian primary care settings, and these rates exceed the expected prevalence of group A streptococcus among adults and children (6). Previous studies have shown that clinical scoring tools have an acceptable specificity and can limit the overprescribing of antibiotics, thereby reducing the emergence of antibiotic resistance and the cost of health care (7, 8).

This review has gathered information relevant to pharyngotonsillitis. The sources of the information provided are reliable to an extent owing to the references that have been cited. Recent literature has been used, and very few sources of old literature have been used where necessary.

In writing this review, answers to the following questions are sought:

1. What is the prevalence of group A streptococcal pharyngotonsillitis among children and adults worldwide?
2. What is the principle of management for antibiotic prescription in primary care clinics?
3. What are the clinical scoring tools to diagnose group A streptococcal pharyngotonsillitis?
4. What are the sensitivity and specificity of the Centor scoring system?
5. What are the treatment and diagnosis of group A streptococcal pharyngotonsillitis in primary care practice?

## Definition

Pharyngotonsillitis (pharyngitis or tonsillopharyngitis) is one of most common upper respiratory tract infections (1). It is an inflammation involving both the pharynx and tonsils most commonly caused by viral or bacterial infection. Pharyngotonsillitis can be classified as acute or chronic depending on the causative agent and the patient’s immune system efficiency (1).

The typical symptoms of pharyngotonsillitis are a sore throat, red and swollen tonsils, white or yellow patches on the tonsils, and fever (9). The patient’s temperature rises to above 38 °C in group A streptococcal pharyngitis (9). Cough and nasal discharge are more symptomatic of viral infections than bacterial infections. Other symptoms may include swollen anterior cervical lymph nodes, headache, painful or difficult swallowing (dysphagia), loss of voice or changes in the voice, abdominal pain in children, and bad breath. Symptoms usually begin one to three days after exposure (Table 1) (9).

## Etiology of Pharyngotonsillitis

Most cases of pharyngotonsillitis are caused by viruses and sometimes occur as a part of the common cold or influenza syndromes (1). The most common viruses that cause uncomplicated pharyngitis are adenoviruses, especially in children (10). Rhinoviruses cause about 20% of pharyngitis cases, and there are more than 100 serotypes of rhinovirus (10). In addition, pharyngotonsillitis is common in patients with influenza A and constitutes about 50% of the cases, whereas the proportion is lower in patients with influenza B (11). The Epstein-Barr virus is responsible for 19% of pharyngotonsillitis cases and usually spreads from adults to infants (11). Moreover, coxsackieviruses and echoviruses are common enteroviruses that cause pharyngitis (11). Cytomegalovirus, parainfluenza viruses, coronaviruses, and the measles virus may cause pharyngotonsillitis (1, 11). Bacteria cause about 15%–30% of pharyngotonsillitis cases (12). The most common and important bacteria that cause acute pharyngotonsillitis belong to *Streptococcus pyogenes* (group A beta-hemolytic streptococcus). *Streptococcus pyogenes* is responsible for about 15%–30% and about 5%–10% of acute pharyngotonsillitis cases in children and adults, respectively. It is characterised by inflammation, exudates, fever, leukocytosis and tender cervical lymph nodes (1, 12). Groups C and G beta-hemolytic streptococci are a normal flora of the human upper airway, but they can cause pharyngotonsillitis (13). Occasionally groups B, and F beta-hemolytic streptococci are responsible for pharyngotonsillitis. *Haemophilus influenzae* cause pharyngotonsillitis in children less than five years old. Others bacteria such as *Moraxella,* Chlamydia pneumoniae*, Mycoplasma pneumoniae, Corynebacterium diphtheriae*, and *Neisseria gonorrhoeae* cause pharyngotonsillitis, rarely (14, 15)*. Candida albicans* rarely cause pharyngitis when the normal flora is killed through antibiotics therapy or when the individual is immunosuppressed by disease or drugs (Table 2) (14). Table 3 shows the prevalence of group A pharyngotonsillitis among children and adults. The studies included developing and developed countries around the world. The prevalence of group A pharyngotonsillitis around the world varies from 0% to 42.2%.

**Figure 1:**
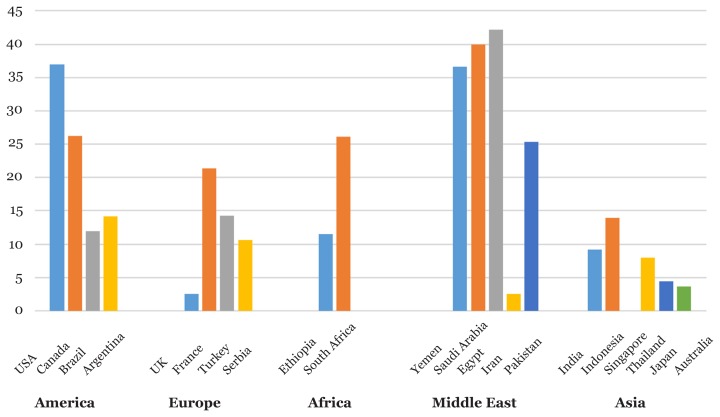
The prevalence of group A pharyngotonsillitis among children and adults worldwide

Figure 1 shows the wide variation in prevalence of group A pharyngotonsillitis around the world. The highest prevalence of group A pharyngotonsillitis was recorded in the Middle East, while the studies in the Southeast Asian countries has reported the lowest the prevalence. The rate of infection was higher among children than adults. These variations in the prevalence of group A pharyngotonsillitis between the different countries due to several factors such as the study design, sampling technique, cultural, ecological, and others (37). Furthermore, the incidence pharyngotonsillitis is more common in children, especially in school age because they are in close contact with the pathogens, where infection spreads through close contact with an infected child, when a person inhale droplets of respiratory secretions from the air when an infected person breathes, coughs, or sneezes (38). An environment, polluting the environment and the lack of ventilation and air pollution, all of these environmental factors reinforce the spread of infection (14). In addition, the peak incidences of pharyngotonsillitis occur in the winter and spring with sporadic cases throughout the year (38). Other risk factors for pharyngotonsillitis are sickle cell anemia, sinusitis, smoking or exposure to secondary smoke, and any condition that weaken the immune system, such as diabetes, organ transplant, chemotherapy, and AIDS (38). All racial and ethnic groups are affected by pharyngotonsillitis worldwide and the infection can affect both male and female genders (39).

## Physical Examination

Majority of physicians depends on the clinical findings to diagnose pharyngotonsillitis but cannot distinguish between streptococcal from non-streptococcal infections according to the clinical findings (40, 41). In addition, signs and symptoms of pharyngotonsillitis overlay extensively with other infectious, making the diagnosis according only to the clinical findings is inaccurate (40). Pharyngotonsillitis is manifested by white or gray patches on the tonsils, swelling of the tonsils, and redness, and inflammation of pharyngeal wall. Usually there will be swollen anterior lymph nodes in the sides of the neck with group A streptococcus pharyngotonsillitis. In addition, ear and nose inflammation might be occurred where pharyngotonsillitis conferred an increase conferred an increase of ear and nose infections. Spreading of bacterial infections from pharynx to the nose and middle ear because they are located in the same cavity might be an explanation of this association (42).

## Laboratory Diagnosis

**Figure 2:**
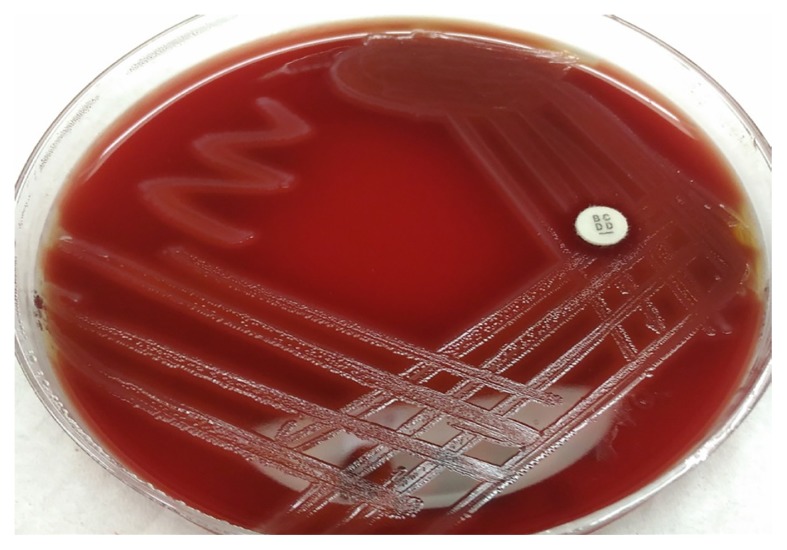
Appearance of *Streptococcus pyogenes* produces a zone of β hemolytic around the colonies and it is sensitive to bacitracin disc on the blood agar, following 24 h of incubation under anaerobic conditions

The gold standard laboratory diagnosis of streptococcal pharyngotonsillitis is culture of throat swab. Culture of throat swab is a laboratory diagnostic test to evaluate the presence of a streptococcal infection in the throat (43). The sample is collected from the tonsils and the wall of pharynx by swab. Presence of *Streptococcus pyogenes* onto blood agar confirm the streptococcal pharyngotonsillitis (Figure 2). The results are further identified based on biochemical test such as Gram stain and Pyrrolidonyl arylamidase test (43). The sensitivity and specificity of the throat swab cultures (81.1%, and 94.9%, respectively) are more than the sensitivity and specificity of the rapid antigen detection test (76.8%, and 93.8%, respectively) (44). However, the throat swabs culture is not effective enough because of the relatively long time (two to three days) for the bacterial growth to be identified (43).

Rapid antigen detection test (RADT) can be an appropriate alternative test to identify group A streptococcus directly from throat swabs because it is a rapid test and takes few minutes in providing results as compared to throat swab culture. Therefore, the physician can make a decision about the therapy at the point of care (45). According to previous studies, rapid antigen detection test has been hindered because it has low sensitivity (46). Therefore, the Centres for Disease Control and Prevention (CDC) and American Academy of Family Physicians (AAFP) guidelines have recommended that the negative RADT results should be backed up by a swab throat culture to increase the accuracy of the diagnosis (47). However, RADT has a high specificity, this means that the positive RADT results do not require to be backed up by a swab throat culture (48). More recently, molecular based techniques, such as DNA probes and polymerase chain reaction (PCR) have been developed to identify the pathogens that cause pharyngitis. It is a sensitive, and specific method, but it is not used routinely in most of the countries around the world, and usually it is used in epidemiological studies (49). Anti-streptolysin-O (ASO) test is a rapid latex agglutination test to determine anti-streptolysin-O antibodies in patient’s serum who had a recent β-haemolytic streptococci infection. The titer increases after 7 days of infection and reach the peak after 4 to 6 weeks (50).

## Clinical Tools to Diagnose Group A Streptococcal Infection

Clinical tools are a guideline for a quick diagnosis and treatment of group A streptococcal pharyngotonsillitis in patients with complaints of sore throat (51). It is a screening method to help physicians determine if the patient needs testing (throat culture or rapid antigen detection test) or not and if the patient needs empiric antibiotic therapy (52).

All the studies that reported the clinical prediction rules for diagnosing group A streptococcal pharyngotonsillitis among adults and children were eligible to inclusion for the purpose of this review. The clinical prediction rules were defined as a decision-making tool that included two or more variables obtained from the physical examination or the history of the patients. All studies that updated by the original authors only was included (Table 4). Most of these tools are clinical scores based on the clinical findings which is associated with group A streptococcal infection. Previous studies regarding to the accuracy of diagnosis of group A streptococcal pharyngotonsillitis by using these tools have shown that, most of these strategies did not show sufficient accuracy, and were unable to identify patients in a low or high-risk case. However, several studies have shown that Centor and MacIsaac scoring have a sensitivity more than 80% and a specificity more than 60% (8, 53).

## Centor Scoring System

In 1981, Centor et al. (55) have developed set of four criteria which might be used to identify the likelihood of group A streptococcal infection in adult patients complaining of a sore throat. Centor criteria including the presence of fever equal to or more than 38 °C, absence of cough, swollen anterior cervical lymph nodes, and tonsillar exudates or swelling, one point is added for each criterion. The Centor scores might range from 0–4 (Table 5) (55).

**Figure 3:**
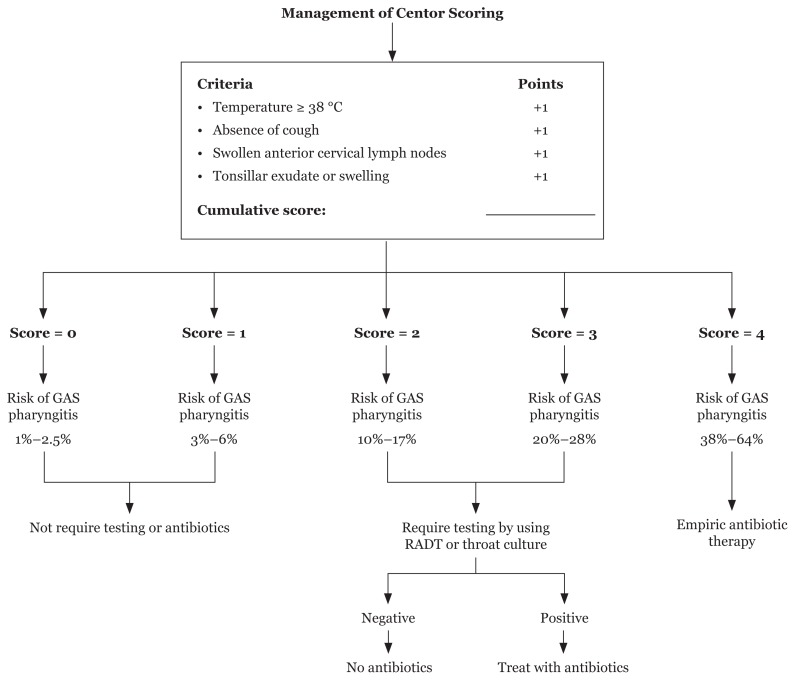
Centor scoring management

A patient with a score of zero or one do not require testing or antibiotics because the risk of streptococcal pharyngitis is very low between 2% and 6%, but a patient with a score of two or three is require testing by using rapid antigen detection test or throat culture, because the chance of streptococcal infection is around 10%–28% and require anti-microbial therapy only if the result is positive. However, a patient with a score of four should be tested by using rapid antigen detection test or throat culture, and empiric antibiotic therapy is required due to chance of streptococcal infection is high between 38% and 64% (Figure 3) (61).

Previous studies about the accuracy of Centor criteria have shown that, the sensitivity of absence of cough and swollen anterior cervical lymph nodes was higher than the specificity (sensitivity 76%, specificity 48% and sensitivity 68%, specificity 58%, respectively), while the specificity of the presence of fever (≥ 38 °C) and tonsillar exudates was higher than sensitivity (sensitivity 51%, specificity 71% and sensitivity 58% and specificity 75%, respectively (8). In addition, the positive likelihood ratio (LR) of tonsillar exudates was the highest positive likelihood ratio equal 2.20, suggesting it raises the probability of having group A pharyngotonsillitis between 15% and 20%, while the positive likelihood ratio (LR) of fever (≥ 38 °C) was 1.67 and it raises the probability of having group A pharyngotonsillitis between 10% and 15% when present. Absence of cough and swollen anterior cervical lymph nodes both decrease the likelihood of having group A pharyngotonsillitis between 15% and 20% when absent (8). The presence of all four criteria have a positive predictive value between 40% and 60% for group A streptococcal pharyngotonsillitis. However, the absence of all criteria have a negative predictive value more than 80%. Therefore, these studies have suggested that the presence of all four criteria discriminates group A streptococcal pharyngotonsillitis from other etiology in patients with sore throat (8).

Furthermore, studies have shown that the Centor score has an acceptable sensitivity and specificity (sensitivity 81%, and specificity 70%, respectively) in predicting likelihood of group A streptococcal pharyngotonsillitis. Applying this to prescribing decision, can limit the over prescription of antibiotics, thus will reduce the emergence of antibiotic resistance as well as the cost of health care but should be used in primary care clinics. However, laboratory confirmation is the gold standard in making an accurate diagnosis (7). In addition, Centor scoring provides quick diagnosis of group A streptococcal pharyngotonsillitis in adult patients, unlike the tests to confirm a diagnosis of streptococcal pharyngotonsillitis, such as a culture which takes two to three days for the results in order to avoid any complications (62).

## MacIsaac Scoring System

MacIsaac score are similar to Centor score, but it adds one extra point for patient less than 14 years old, because this age group is more susceptible to streptococcal pharyngotonsillitis and subtract one point if the patient age 45 years old or more. Therefore, the score might range from −1 to 5. A low score on MacIsaac criteria help to exclude streptococcal pharyngotonsillitis, and higher scores require testing by using rapid antigen detection test or throat culture, and empiric antibiotic therapy (7, 53).

## Treatment of Pharyngotonsillitis

### Bacterial Pharyngotonsillitis Treatment

Bacterial pharyngotonsillitis is treated by anti-microbials. Penicillin, a course of oral penicillin V for 10 days or a single dose of parenteral penicillin G are the best choice to treat group A streptococcal pharyngitis. However, amoxicillin can be used. In penicillin allergic patients, cephalosporins, clindamycin, erythromycin or azithromycin are an acceptable alternative (5). In addition, group C and G streptococcal pharyngitis are usually treated with antibiotics, although it lacks the clinical trials (5, 63).

To help physicians to prescribe the appropriate antibiotics for children and adults with streptococcus pharyngitis, Centres for Disease Control and Prevention (CDC), American Academy of Family Physicians (AAFP), and the Institute for Medical Research (IMR) have developed the clinical practice guidelines for respiratory tract infections (47, 64, 65). Moreover, the guidelines were compiled by the leading medical organisations (47, 65).

According to the CDC, AAFP guidelines, and the National Antibiotic Guideline in Malaysia, antibiotics should be prescribed only to patients with group A streptococcal infection (*Streptococcus pyogenes)* which group A streptococcus usually cause symptoms including sore throat, fever more than 38 °C, exudates, nausea, vomiting, headache, stiff and swollen neck, abdominal pain, and tender enlarged cervical lymph nodes. However, cough, nasal discharge, conjunctivitis, and diarrhea are more symptomatic of viral infections than group A streptococcal infections and the viral infections do not treat with antibiotics. The diagnosis must be confirmed by throat culture or rapid antigen detection test (47, 64, 65).

CDC and AAFP guidelines recommended antibiotics for patients with sore if the patient is younger than 6 months, diagnosis certain, or in severe illness (66). The first-line of antibiotics to treat group A streptococcal infection in children and adults are oral penicillin V for 10 days or a single dose of parenteral penicillin G; however, the alternative therapy for children are amoxicillin, oral cephalosporins, clindamycin, and macrolides. Amoxicillin, macrolides, erythromycin, oral cephalosporins, clindamycin in adult patients allergic to penicillin for 10 days. Analgesics and antipyretics such as ibuprofen or acetaminophen can be used to reduce the pain (67).

The National Antibiotic Guideline in Malaysia has recommended amoxicillin 500 mg or phenoxymethylpenicillin 500 mg for 10 days to treat pharyngotonsillitis that are caused by group A streptococcus; however, the alternative therapy is a single dose of benzathine penicillin, azithromycin 500 mg for 5 days or clindamycin 300 mg–450 mg for 10 days to patients who allergic to penicillin (65).

### Other Treatment

Viral pharyngotonsillitis is treated by a symptomatic relief, such as soothing fluids, aspirin, paracetamol, soothing gargle, and rest with drinking an adequate water and hot fluid (68). Tonsillectomy or surgery to remove the tonsils is indicated when complications of pharyngotonsillitis (repeated occurrence of acute tonsillitis, peritonsillar abscess or obstruction of the nasal airway) (69). Anti-inflammatory medications such as aspirin for pain or corticosteroids might be required if the complications of group A streptococcal pharyngotonsillitis such as rheumatic fever occur (4).

## Antibiotics Practices in Primary Care Clinics

Anti-microbial resistance is driven by many factors, the most significant of which is inappropriate prescribing. This is when patients get a prescription for an antibiotic that they don’t really need, or get a prescription for the wrong antibiotic, the wrong dose or the wrong duration. Typically, antibiotics are prescribed by physicians to the patients based on clinical symptoms. There are reasons for inappropriate prescriptions are very difficult to curb, including the difficulty of diagnosis of whether a viral or bacterial infection to determine the appropriate treatment and it is known antibiotics can only be given to patients with bacterial infection (70). Patients play an important role in that issue, because most of the patients insist that physicians to prescribe the antibiotic and that increase demand for a group of antibiotics speeds up the resistance of antibiotics and it becomes useless (70).

The antibiotic prescription rate in a study at public primary care clinics which was carried out in Malaysia was 34.1% among patients with sore throat (71). A study that carried out in Jordan among patients with sore throat has reported that antibiotics were prescribed for 78.4% of patients with sore throat (72). Another study done by Lauri et al. in the United States of America has reported that the rate of prescribed antibiotics to adults with sore throat was 73.7% (73).

The Centres for Disease Control and Prevention (CDC), American Academy of Family Physicians (AAFP), and the National Antibiotic Guideline in Malaysia strongly recommend the strategies of Centor scoring to select patients need for antibiotic therapy. The goal of the strategies is to limit unnecessary antibiotic prescribing. These strategies are expected to limit the antibiotic prescribing rates between 10% and 33%, lower than current rates which is more than 65%. In addition, the strategies are expected to administer appropriate analgesics, anti-pyretics and supportive care of patients with pharyngotonsillitis (64, 65).

The Infectious Diseases Society of America (IDSA) and American College of Physicians (ACP) guidelines, conversely, have recommended that the diagnostic testing is required (RADT or throat culture in all patients at risk) and cannot depend on the clinical diagnosis of GAS pharyngotonsillitis alone. IDSA and ACP guidelines recommend confirmatory for negative RADT by throat swab culture in children (74). In addition, according to the United Kingdom National Health Service (UKNHS), the antibiotic is prescribed only to high-risk and very ill patients (52).

## Anti-microbial Sensitivity and Resistance Patterns

Group A streptococcus (*Streptococcus pyogenes*) and other beta haemolytic streptococci are sensitive to penicillin and cephalosporin as well as rifampin and vancomycin. However, some strains of the bacterium have been found to be resistant to macrolides, tetracycline, and clindamycin (75).

The excessive take of unnecessary antibiotics led to increase and spread the resistant of anti-microbial in communities around the world. The increasing of anti-microbial resistance has been attributed to combinations of microbial characteristics, selective pressures of anti-microbial use, and societal and technological changes that enhance the transmission of drug-resistant organisms (76). Furthermore, the resistance of anti-microbial lead to increase the morbidity and mortality since resistance increases the risk of inappropriate therapy. The risk is that the patients who do not receive appropriate therapy will have a longer period of disease or fatal effect; moreover, morbidity and transmission of the microorganism will increase due to the patients remain infectious for a long period. Increased morbidity and mortality was documented in several cases including streptococcal infection (77). A study in France has estimated that 158,000 infections due to multidrug-resistant microorganisms occurred in 2012, including 12,500 deaths were associated with these infections (77). In addition, another study in 31 European countries has reported that 27,711 infections due to multidrug-resistant microorganisms were associated with 5,503 deaths (78).

In term of cost, the cost of the consequences of anti-microbial resistance might include hospitalisation for long period, costs of additional laboratory testing and including full health care costs. The costs of antibiotic therapy depend on the dose and duration of treatment and often are expensive due to there are few antibiotics available to treat the multidrug-resistant bacteria. In addition to this costs, the costs of the efforts and studies to eradicate the anti-microbial resistance from health care centres. The costs of morbidity and mortality has indirect effects and are not easy to be measured and economically evaluated due to a number of effects such as extension of resistant genes between different species and strains (79). However, the morbidity, mortality and economic impact of streptococcal infection has not been elucidated (7, 79).

Previous studies have shown that there was a high rate of resistance among group A streptococcus and other beta haemolytic streptococci to antibiotics β-lactam, but they were highly susceptible to penicillin vancomycin, ofloxacin, cephalosporin, and clindamycin (75). In addition, another study in South Korea have reported that the resistance to erythromycin and clindamycin has markedly increased during the period 1995 to 2002 (80). In Malaysia, according to the Institute for Medical Research (IMR), for group A streptococcus, erythromycin resistance was 5.7% in 2012 and has decreased to 5.4% in 2013. The resistant to clindamycin has increased to 4.4% in 2013 compared to 3.9% in 2012. Tetracycline resistance in 2013 has increased to 58.4% compared to 55.6% in 2012. For group B streptococci, the resistance to tetracycline, co-trimoxazole and clindamycin have increased in 2013 compared to 2012. Ceftriaxone resistance was less in 2013 (1.3%) compared to 1.7% in 2012 (65).

## Conclusion

The above review has documented that the over prescribing of antibiotics is a global problem. Since pharyngotonsillitis is one of the common reasons for visiting care clinics worldwide, over-prescription of antibiotics reason to occur the anti-microbial resistance. Although, there is a lack of the data on the above matters, the available studies indicated that, there is an urgent need to reduce the over-prescription of antibiotics in the health care centres and clinics. One of the solutions is to follow the strategies that help to diagnose group A streptococcal infection. According to previous studies, the clinical rules for triaging patients who should undergo group A streptococcal testing was poor, however, Centor and MacIsaac scoring have shown high sensitivity and acceptable specificity. Further validation studies about the clinical prediction rules are needed to ensure the accuracy and update the clinical strategies. Future studies should also aim to detect the accuracy of diagnosis using the rapid antigen detection testing and the gold standard to diagnose group A streptococcal pharyngotonsillitis.

## Figures and Tables

**Table 1:** Clinical and epidemiologic characteristics of group A streptococcal (GAS) pharyngotonsillitis and viral pharyngotonsillitis (9)

GAS pharyngotonsillitis	Viral pharyngotonsillitis
Sudden onset	Conjunctivitis
Sore throat	Sore throat
Fever ≥ 38 °C	Cough
Scarlet fever rash	Nasal discharge (rhinorrhea)
Nausea, vomiting, and abdominal pain	Loss of voice or changes in the voice
Headache	Diarrhea
Swollen anterior cervical lymph nodes	Myalgia
Tonsillar swelling or exudates	
Patient aged 5–15 years	
History of exposure	

**Table 2:** Etiology of pharyngotonsillitis (14)

Etiologic Agent	Clinical Findings
**Bacteria**	
*Streptococcus pyogenes*	Scarlet fever, enlarged anterior cervical nodes, and fever ≥ 38 °C
Groups C and G Streptococci	
*Neisseria gonorrhoeae*	
*Corynebacterium diphtheriae*	Diphtheria
*Mycoplasma pneumoniae*	Pneumonia
*Haemophilus influenzae*	Children less than 5 years old
**Viral**	
Rhinovirus	Common cold
Adenovirus	Pharyngoconjunctival fever and acute respiratory disease
Influenza virus	Common cold and rhinorrhea
Coronavirus	Common cold
Herpes simplex virus types 1 and 2	Gingivostomatitis
Parainfluenza virus	Common cold and croup
Epstein–Barr virus	Infectious mononucleosis
**Candida**	
*Candida albicans*	Slight sore throat and hoarseness

**Table 3:** The prevalence of group A pharyngotonsillitis among children and adults worldwide-studies published since 2001

Author	Country, Year	Prevalence (%)	Population or subject (*n*)	Setting
**America**				
Shaikh et al. (16)	USA, 2010	37%	266 (children)	Centres for Disease Control and Prevention’s Active Bacterial Core surveillance (ABCs)
Bocking et al. (17)	Canada, 2016	26.2%	6674 (all ages)	Health centres
Tartof et al. (18)	Brazil, 2010	12%	2194 (children)	Two pediatric outpatient clinics
Delpech et al. (19)	Argentina, 2017	14.2%	127 (children)	Pediatric outpatient clinic
**Europe**				
Ogle et al. (20)	UK, 2012	2.2%	600 (children)	Pediatric emergency department and two pediatric outpatient clinics
Humières et al. (21)	France, 2015	21.4%	585 (children)	Hospital outpatient clinics
Durmaz et al. (22)	Turkey, 2003	14.3%	1419 (children)	Three primary schools
Mijač et al. (23)	Serbia, 2010	10.6%	145 (all ages)	Health centres
**Africa**				
Tesfaw et al. (24)	Ethiopia, 2015	11.5%	355 (children)	Health centres
Engel et al. (25)	South Africa, 2014	26.1%	742 (all ages)	Hospital outpatient clinics
**Middle East**				
Ba-Saddik et al. (26)	Yemen, 2014	36.6%	730 (children)	Eight schools
Telmesani et al. (27)	Saudi Arabia, 2002	40.0%	73 (children)	Four schools
Abd El-Ghany et al. (28)	Egypt, 2015	42.2%	142 (children)	Pediatric outpatient clinic
Khosravi et al. (29)	Iran, 2016	2.5%	1000 (children)	Children’s hospital
Rathi et al. (30)	Pakistan, 2014	25.3%	5140 (all ages)	Eight primary care clinics
**Asia**				
Trupti et al. (31)	India, 2016	9.2%	218 (all ages)	Public health centres
Syahroel et al. (32)	Indonesia, 2008	14%	95 (children)	Hospital pediatric clinic
Hong et al. (33)	Singapore, 2004	0.0%	594 (adults)	Public health centres
Treebupachatsakul et al. (34)	Thailand, 2006	8.0%	292 (adults)	Hospital outpatient clinic
Karasawa et al. (35)	Japan, 2001	4.4%	360 (children)	Primary care clinics
McDonald et al. (36)	Australia, 2006	3.7%	294 (children)	Primary care clinics

**Table 4:** The clinical criteria to selectively test for group A streptococcal infection

Clinical screening rule	Study location and period	Criteria	Suggestions from the study
Forsyth (54)	USA, 1975	Fever, enlarged tender nodes, exudate, the laboratory results for white blood cells, mucoid exudate, multiple small nodules and myalgia	Clinically non-streptococcal: culture; treat symptomatically Clinically streptococcal: do not culture; oral penicillin therapy
Centor (55)	USA, 1981	Fever ≥ 38 °C, absence of cough, tender anterior cervical adenopathy, tonsillar swelling or exudate	0–1: do not require testing or antibiotics 2–3: rapid antigen detection test or throat culture, antibiotic therapy if the result is positive 4: rapid antigen detection test or throat culture, and empiric antibiotic therapy
Edmond (56)	Australia, 1996	Age, scarlatiniform rash, erythema, swollen tonsils, edematous, and tender cervical nodes	< 20%: no rapid antigen detection testing, no antibiotic treatment 20%–60%: rapid antigen detection testing, antibiotic with positive result > 60%: no testing, antibiotic therapy
MacIsaac (57)	Canada, 1998	Fever ≥ 38 °C, absence of cough, tender anterior cervical adenopathy, tonsillar swelling or exudate, and age	≤ 0–1: no rapid antigen detection test, no antibiotic required 2–3: culture or rapid antigen detection test, antibiotic if the result is positive ≥ 4: culture and treat with antibiotic
Attia (58)	USA, 2001	Scarlatiniform rash, tonsillar swelling, tenderness and enlargement of cervical lymph nodes, and absence of runny nose	0: no rapid antigen detection testing, or antibiotic therapy 1–3: antibiotic therapy with positive result ≥ 4: no testing, antibiotic treatment
Joachim (59)	Brazil, 2010	Age, tender cervical node, headache, abdominal pain, sudden onset	≤ 2: no rapid antigen detection test, no antibiotic therapy 3: rapid antigen detection testing, antibiotic with positive result ≥ 4: no testing, antibiotic therapy
Suzumoto (60)	Japan, 2009	Presence of sore throat symptoms and the laboratory results for white blood cells and C-reactive protein	Mild cases: not require testing or antibiotics Moderate cases: antibiotic therapy (ampicillin and levofloxacin) Severe cases: parenteral antibiotics therapy

**Table 5:** Centor score criteria (55)

Criteria	Score
Temperature ≥ 38 °C	1
Absence of cough	1
Swollen anterior cervical lymph nodes	1
Tonsillar swelling or exudates	1
